# Anti-melanogenic activity of *Myristica fragrans* extract against *Aspergillus fumigatus* using phenotypic based screening

**DOI:** 10.1186/s12906-020-2859-z

**Published:** 2020-03-02

**Authors:** Shanu Hoda, Maansi Vermani, Rajesh K. Joshi, Jata Shankar, Pooja Vijayaraghavan

**Affiliations:** 1 Amity Institute of Biotechnology, Amity University Uttar Pradesh, Noida, 201301 India; 2 Department of Phytochemistry, National Institute of Traditional Medicine, Indian Council of Medical Research, Nehru Nagar, Belagavi, Karnataka 590010 India; 3 Genomic Laboratory, Department of Biotechnology and Bioinformatics, Jaypee University of Information Technology, Solan, Himachal Pradesh 173212 India

**Keywords:** *Myristica fragrans* spice, *Aspergillus fumigatus*, Melanin biosynthesis, Virulence, GC-MS

## Abstract

**Background:**

*Aspergillus fumigatus*, an opportunistic fungal pathogen is associated with a wide array of diseases. It produces 1, 8-dihydroxy naphthalene (DHN) melanin that imparts greenish grey color to conidia and is an important virulence factor. It masks various molecular patterns associated with *A. fumigatus* and protects the fungus from host immune system. *Myristica fragrans*, enriched with secondary metabolites has been traditionally used for the treatment of infectious and inflammatory diseases. The present study was aimed to explore the anti-melanogenic effect of *M. fragrans* extracts on *A. fumigatus*.

**Methods:**

*M. fragrans* extracts (hexane, chloroform, methanol and ethanol) were prepared through polarity guided extraction. Phytochemical analysis was performed to detect the chemical constituents of the extracts. The minimum effective concentration (MEC) of the extracts against *A. fumigatus* melanin was determined by broth micro-dilution assay*.* Various virulence factors were assayed by spectrophotometric methods. Electron microscopic studies were performed to evaluate the effect of the hexane extract of *M. fragrans* on *A. fumigatus* cell surface morphology. The major active compounds of the extract were detected by gas chromatography-mass spectrometry (GC-MS). Docking was performed to study the interaction between the major identified compounds and the ketosynthase domain of polyketide synthase protein.

**Results:**

The results indicated that the hexane extract of *M. fragrans* inhibited melanin production (76.09%), reduced ergosterol content (83.63%) and hydrophobicity of the cell (72.2%) at the MEC of 0.078 mg/mL. Altered conidial surface, disappearance of protrusions and absence of melanin layer on outer cell surface was observed in electron microscopy. Forty-two compounds were identified by GC-MS. The main constituents were identified as sabinene (12.2%), linoleic acid (11.7%), hexadecanoic acid (10.5%), safrole (8.1%) and elemicin (7.8%). Docking studies revealed that hexadecanoic acid, its derivative compound cis-9-hexadecenal and isoeugenol have lower binding energy forming proper hydrogen bond with ketosynthase domain of polyketide synthase protein.

**Conclusion:**

The study concludes that the extract of *M. fragrans* has potential antifungal properties that can be explored in combination with available antifungals. This combination approach may be helpful for large number of patients suffering with *A. fumigatus* infections.

## Background

*Aspergillus* species are the most prominent airborne fungal pathogens that account for various invasive and non-invasive infections based on the impaired immune system in humans [1, 2]. They propagate by conidia which are ubiquitous and have high sporulating capacity. Once inhaled, they by-pass mucociliary clearance, germinate and produce septate vegetative mycelium that invades the lung tissue [3]. Among *Aspergillus* infections, more than 90% of pulmonary infections are caused by *Aspergillus fumigatus* [4]. Major pulmonary diseases due to *A. fumigatus* include allergic bronchopulmonary aspergillosis (ABPA), *Aspergillus* rhinosinusitis, chronic pulmonary aspergillosis, invasive aspergillosis, saprophytic aspergilloma and trachoebronchitis. These diseases manifestations can coexist in the same individual.

There are limited antifungal drugs available in the market to combat fungal infections. These drugs mainly target fungal cell wall (echinocandins) and ergosterol by either binding to ergosterol (polyenes) or inhibiting intermediary enzymes of ergosterol production (azoles and allylamines). These chemically synthesized drugs have several adverse effects such as anaphylaxis, chills, fever, headache, hepatotoxicities, nausea, neurotoxicity, and reproductive disorder [5]. These classical drugs are not cost effective [6]. Besides, the incidence of drug resistance in *Aspergillus* is increasing globally. Hence, there is an urgent need for affordable antifungal medications to improve the drug efficacy and reduce the side effects. The use of herbal medicines may be a suitable alternative that can open new avenues in antifungal treatment.

Various plant extracts have been reported to exhibit antibacterial, antifungal and insecticidal properties in vitro [7–11]. Spices contain various secondary metabolites such as flavonoids, phenolics, tannins, terpenes, quinines and are associated with antioxidant property. These bioactive compounds are involved in antimicrobial activity by the cell wall disruption, leakage of cellular components, fatty acid and phospholipid alteration [12].

*Myristica fragrans* is a dietary spice; belongs to the Myristicaceae family and is cultivated in the Banda Islands, Grenada, the Caribbean, South India, Sri Lanka, Malaysia, Sumatra, and Brazil [13]. The main constituents of *M. fragrans* have been found to be alkyl benzene derivatives (myristicin, elemicin, safrole etc.), terpenes, alpha-pinene, beta-pinene, myristic acid and trimyristin [14]. It contains about 2% of lignans (diarylpropanoids), which are non volatile dimers of phenylpropanoid [15]. *M. fragrans* has been traditionally used for intestinal catarrh and colic, to stimulate appetite, control flatulence and also as an abortifacient [16, 17]. It has been reported as an antidepressant with antioxidant and hepatoprotective role [18].

Multiple factors contribute to virulence of *A. fumigatus* including its versatile metabolism, thermo-tolerance, or the production of toxins and secondary metabolites like 1,8-dihydroxy naphthalene (DHN) melanin [19]. DHN-melanin plays a major role in the protection of Aspergilli against harsh environmental conditions such as ultraviolet irradiation, reactive oxygen species and the host immune system [20]. Melanin has been shown to provide cell wall stability and structural rigidity [21]. It binds to antifungal drugs reducing their therapeutic efficacy. Time kill assay revealed that 71 and 79% of melanised yeast cells survived on exposure to 2X the MIC of amphotericin B and caspofungin, respectively [22]. Besides, melanin prevents intracellular killing of conidia by reducing luminal acidification and resisting phagolysosomal degradation [1, 3]. The role of the conidial melanin in *A. fumigatus* has been studied by using pksP gene mutants (∆pksP). The pksP gene encodes the first protein polyketide synthase (PKS) that catalyse the synthesis of heptaketide napthopyrone, the first step in the melanin biosynthesis pathway. The ∆pksP produce white color conidia whereas other gene mutants produced yellowish, reddish or brown colonies. It has been observed that melanized conidia masked various *A. fumigatus* associated molecular patterns to protect themselves from elimination whereas, the unpigmented white conidia were unable to quench reactive oxygen species (ROS) produced by human and animal granulocytes and were effectively eradicated by the host defense system [1, 23]. Therefore, restraining the DHN-melanin biosynthesis through pksP gene/gene product inhibition may be considered as a novel drug target. To the best of author’s knowledge, the role of *M. fragrans* as melanin inhibitor in *A. fumigatus* has not been studied till date. Thus, the present study was envisaged to evaluate the efficacy of *M. fragrans* extract as a melanin inhibitor in *A. fumigatus*.

## Methods

### Fungal strains

*Aspergillus fumigatus* (ATCC-46645) and its ∆pksP strain were a kind gift from Prof. Axel Brakhage, Department of Molecular and Applied Microbiology, Lelbeniz Institute for Natural Product Research and Infection Biology- HKI, Germany. *A. fumigatus* strains were maintained by subculturing on Czapek Dox Agar (CzA) slants. The fungus was grown on CzA at 28 ± 2 °C for 5 days to obtain conidial growth.

### Plant collection and extraction procedure

The *Myristica fragrans* dried spice was procured from Spice Garden in Thekkady, Kerala, India and was authenticated by Dr. (Mrs) Sunita Garg, CSIR-Emeritus Scientist (Former Chief Scientist & Head, Raw Materials Herbarium and Museum Delhi, CSIR-National Institute of Science Communication And Information Resources). A voucher specimen has been deposited in Raw Materials Herbarium and Museum Delhi and the deposition number is NISCAIR/RHMD/Consult/2018/3250–51.

Cleaned and dried spice (50 g) was ground using mortar and pestle to make a fine powder. It was then extracted with 200 mL hexane under constant shaking at room temperature for 72 h. Thereafter, the extract was filtered and dried under vacuum using rotary evaporator. Further, the polarity guided extraction was done sequentially using chloroform, methanol and ethanol to yield hexane (8.54 g), chloroform (5.78 g), methanol (2.88 g) and ethanol (1.88 g) dried extracts. The dried extracts of *M. fragrans* were re-suspended in dimethyl sulfoxide (DMSO) to make the stock suspension of 100 mg/mL and stored at 4oC for further experiments. The prepared extracts were coded as JaH, JaC, JaM and JaE extract, respectively.

### Phytochemical analysis of extracts

Preliminary qualitative phytochemical analysis of the *M. fragrans* extracts was carried out to detect the presence of saponins, tannins, terpenoids, carbohydrate, steroids, napthaquinones and flavonoids as per the protocol of Raaman [24].

### Determination of minimum effective concentration (MEC) of extracts for demelanization

Minimum effective concentration with respect to the appearance of white colonies was determined by Clinical and Laboratory Standards Institute (CLSI) broth micro-dilution method [25]. The experiment was carried out in triplicates. Two-fold serial dilutions of the extracts were made in Czapek Dox Broth (CzB) over a range to give final concentrations of 5.0–0.009 mg/mL. One hundred microliters of *A. fumigatus* conidial suspension 0.8 × 104 conidia/mL was added to each well. Negative control comprised CzB only while the positive control was CzB and conidial suspension. The final volume in each well was 200 μl. The MECs of the extracts were detected after 5 days incubation at 28 ± 2 °C. MEC was defined as the lowest concentration of the extract that produced white colored conidia based on morphological appearance [25].

### Extraction, purification and UV- visible spectrophotometric analysis of melanin

The isolation of cell-wall associated melanin from wild type conidia and treated conidia was performed as described by Rajagopal et al. [26]. The cultures were grown in CzA plates for 5 days at 28 ± 2 °C. The treated culture plates were supplemented with minimum effective concentration of the hexane extract (JaH). Positive control plates were also prepared. Mycelial plug (1 cm diameter) was cut from the colonies grown on CzA, boiled in 5 mL of distilled water for 5 min and then centrifuged for 5 min at 7000 rpm. The pellet was washed twice and melanin was extracted by autoclaving the pellet with 3 mL of 1 M KOH. The extracted melanin was dried overnight at 20 °C in a dehumified atmosphere. Further, acid hydrolysis was done to purify the extracted melanin by adding 5 mL of 7 M HCL in a sealed glass vial for 2 h at 100 °C. After cooling, the pigment was washed thrice with distilled water, dried, and stored at 4 °C. Extracted and dried pigment from solid culture was dissolved in 1 M KOH and melanin was recorded as the absorption spectra at range of 200–600 nm using 1 M KOH as reference blank. The absorption spectra of the control and treated plates were determined and compared with the ∆pksP strain.

Melanin pigment isolated from the culture supernatant was assayed by harvesting the cells from culture broth by centrifugation. The pellet was dissolved in 1 M KOH and was vortexed properly. It was centrifuged at 7000 rpm for 5 min and the supernatant were purified by adding 7 M HCl and boiled for 2 h, centrifuged and pellet was washed with distilled water. Extracellular melanin was assayed by dissolving the pellet in 1 M KOH and measuring the absorbance at 585 nm [27].

### Analysis of physio-chemical properties of melanin

*A. fumigatus* was cultured on CzA with JaH extract (at MEC) and without the extract. The color patterns of the colonies were compared with the ∆pksP strain. The pigments extracted from the isolates were confirmed as melanin on the basis of their physical and chemical properties. Characterization of melanin is based on the solubility in NaOH/ KOH, insolubility in water or organic solvents, decolorization by the oxidizing agents (H2O2/ KMnO4) and precipitation by 1% FeCl3 [28].

### Effect of JaH extract on conidia production in *A. fumigatus*

The reduction in conidia formation was estimated spectrophotometrically at 530 nm. Agar blocks (1 cm3) was excised from the 5 day old CzA plate using a sterile surgical blade and transferred to test tube. Phosphate buffer supplemented with 0.25% Tween-20 (5 mL) was added to each tube, shaken vigorously and absorbance was measured at 530 nm. The absorbance of control and the treated conidia were compared with respect to the ∆pksP strain.

### Ergosterol content estimation

Ergosterol content was measured as described by Young et al. with minor modification [29]. Briefly, 5 days old mycelia were harvested by centrifugation at 2700 rpm, washed with sterile distilled water, dried and weighed. The dried pellet was transferred into sterile glass screw cap tubes and 3 mL of 25% of alcoholic KOH solution was added. The pellets were then incubated at 85 °C in water bath for 1 h. Tubes were kept at room temperature. Further, 1 mL of distilled water and 3 mL of *n*-octane was added, vortexed for 3 min and then allowed to stand for separating the organic layer, which was transferred to another clear screw cap tube and stored at − 20 °C. For analysis, 200 μL of the extracted sterol was mixed with 800 μL absolute ethanol and was spectrophotometrically analyzed at 281.5 nm and 230 nm. The conversion from optical density to ergosterol content was calculated as follows [29]:

$$ \mathrm{Ergosterol}\%=\left[\left({\mathrm{A}}_{281.5}/290\ \mathrm{x}\ \mathrm{F}\right)/\mathrm{Weight}\ \mathrm{of}\ \mathrm{pellet}\right]-\left[\left({\mathrm{A}}_{230}/518\ \mathrm{x}\ \mathrm{F}\right)/\mathrm{Weight}\ \mathrm{of}\ \mathrm{pellet}\right] $$

F = Ethanol Dilution Factor

### Cell surface hydrophobicity

Hydrophobicity of the microbial cell suspension was assayed by two phase partitioning using hexadecane as the hydrocarbon phase described by Kennedy et al., with minor modification [30]. Briefly, conidia were harvested using phosphate buffer saline (PBS) and their absorbance was set to 0.30 at 630 nm. Hexadecane (500 μL) was then added and vortexed for 2 min. The suspension was incubated for 10 min at room temperature for phase separation. The absorbance of the aqueous phase was then measured at 630 nm and was compared to the initial absorbance.

### Scanning electron microscopy (SEM)

Conidia from 5 days old *A. fumigatus* cultures grown on CzA medium with and without JaH extract (at MEC) were harvested as described in earlier section. The conidia were fixed in 4% glutaraldehyde in PBS under vacuum for 24 h. After washing, the cells were post-fixed with 1% osmium tetroxide for 60 min and dehydrated by passage through ethanol solutions of increasing concentration. The sample were then mounted on aluminium sheet and coated with gold-palladium alloy. The observations were made using a Zeiss SEM, MA EVO − 18 Special Edition [20].

### Transmission electron microscopy (TEM)

*A. fumigatus* culture was grown in CzA medium with and without the JaH extract for 5 days. Conidia were harvested as described in earlier section, washed in and fixed overnight at room temperature with 2.5% glutaraldehyde with 0.1 M sodium cacodylate buffer (pH 7.4). Conidia were incubated for 1.5 h at 20 °C in a solution of 4% formaldehyde- 1% glutaraldehyde in 0.1% PBS and then incubated in 2% osmium tetraoxide for 1.5 h. Dehydration was accomplished by serial washing in graded ethanol solutions of 50–95% for 10 min, followed by two final washes in 100% ethanol for 15 min. The cells were embedded in Spurr’s resin, sectioned onto nickel grids and examined on a JEOL 2100F transmission electron microscope to obtain micrographs [31].

### Chromatographic analysis

The chemical constituents of the hexane extract of *M. fragrans* were analyzed using gas chromatography varian-450 fitted with a fused silica capillary column TG-5 (30 m × 0.25 mm i.d., 0.25 μm film thickness) [32, 33]. The oven temperature was programmed from 60 to 220 °C at 3 °C/min, using nitrogen as carrier gas. The injector and the flame ionization detector (FID) temperature were set at 230 and 240 °C, respectively. GC–MS analysis was employed using a Thermo Scientific Trace Ultra GC interfaced with a Thermo Scientific ITQ 1100 mass spectrometer which was fitted with a ZB-5 fused silica capillary column (30 m × 0.25 mm; 0.25 μm film thickness). The oven temperature range was programmed from 60 to 220 °C at 3 °C/min and helium was used as carrier gas at 1.0 mL/min for analysis. The injector temperature was set at 230 °C, and the injection volume was 0.1 μL in *n*-hexane, with a split ratio of 1:50. MS was taken at 70 eV with a mass range of m/z 40–450 [34, 35]. Identification of constituents was done on the basis of retention index (RI), determined with reference to homologous series of *n*-alkanes C_8_–C_25_ under identical experimental conditions, by comparing with the MS literature data [36] and co-injection of commercial samples from Sigma-Aldrich, India (≥ 98% purity). The relative amounts of individual components were calculated based on the GC peak area (FID response) without using a correction factor.

### Docking study through protein ligand interaction

Molecular docking study was carried out with AutoDock 4 for ketosynthase (KS) domain of polyketide synthase (PKS) enzyme/protein. The KS domain was searched in NCBI Conserved Domain Search tool and then domain location in the protein sequence was found using FASTA. The Structure of KS was retrieved using software SWISS MODEL (Fig. 6b). The structure of compounds (ligand molecules) was retrieved from PubChem (https://pubchem.ncbi.nlm.nih.gov/; Fig. 6a). The Lamarckian genetic algorithm was used in AutoDock 4 to perform the automated molecular dockings with default parameters. The number of run was set to 50 and the lowest binding energy conformation was selected for LigPlot analysis.

### Statistical analysis

The statistical analysis was conducted using one-way ANOVA to compare the results of melanin estimation, conidiation, CSH and ergosterol assay for extract treated culture with wild type, drug treated and *∆pksP* strain. All experiments were conducted in technical and biological triplicates. The statistical analysis was performed using Graphpad Prism software 8.0.2.263 version and Microsoft Excel. *p* < 0.05 was considered statistically significant.

## Results

### Phytochemical analysis of extracts

The phytochemical tests revealed the presence of steroids, carbohydrate, tannins, alkaloids and terpenoids in the *M. fragrans* extracts (Table 1). However, flavonoids and saponins were not present. JaH extract showed the highest amount of steroids, tannins and terpenoids which were evaluated on the basis of the color production in the respective phytochemical test.

**Table 1:** Phytochemical screening of *M. fragrans* extracts

Name of extract	Tannins	Terpenoids	Flavonoids	Alkaloids	Steroids	Saponins	Carbohydrate
*JaH*	+ +	+ +	–	+ +	+ + +	–	+
*JaC*	+	+	–	+	+ +	–	+
*JaM*	+	+	–	+ +	+ + +	–	++
*JaE*	+ +	+	–	+	+	–	+ +

(+), (+ +) and (+ + +) denotes presence of less, moderate and high amount of phytochemicals respectively, on the basis of color produced during the reaction. (−) denotes absence of phytochemicals. *JaH* Hexane extract of *M. fragrans*, *JaC* Chloroform extract of *M. fragrans*, *JaM* Methanol extract of *M. fragrans*, *JaE* Ethanol extract of *M. fragrans*

### Determination of minimum effective concentration

White colonies of *A. fumigatus* were observed in the wells containing the extract whereas control wells showed greenish grey conidia. The minimum effective concentration was determined as the lowest concentration of the *M. fragrans* extracts showing pigmentless conidial growth as compared to greenish grey wild type conidia. The JaH extract was effective for melanin inhibition at 0.078 mg/mL concentration whereas other extracts inhibited melanin formation at higher concentration. The MEC for JaC and JaM was 1.25 mg/mL and for JaE was 2.50 mg/mL. Therefore, JaH extract was selected for further experiments.

### UV- visible spectrophotometric analysis of melanin

**Fig. 1:**
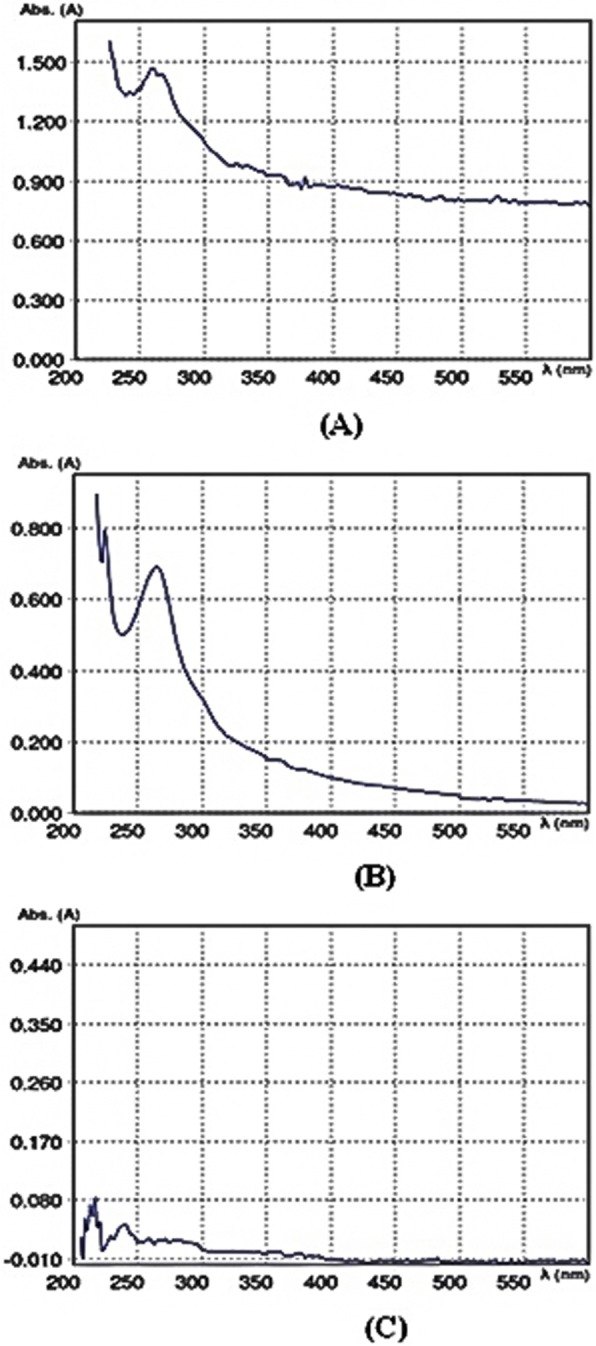
UV-Vis Spectrum of melanin. The melanin was isolated from wild type *A. fumigatus* supplemented with 0.078 mg/mL JaH extract, without extract treatment as well as ∆pksP strain. The melanin obtained from wild type (**a**) and JaH extract treated *A. fumigatus* (**b**) shows typical peak observed in UV range, gradually decreasing with the increase in wavelength whereas, no such characteristic peak observed in ∆pksP strain (**c**)

Melanin was extracted from wild type, JaH extract treated cultures and ∆pksP strain. The spectral study of melanin from both wild type and treated cultures showed a characteristic peak in the UV region 200–260 nm (Fig. 1). The light absorbed by melanin was maximum in the UV region and decreased gradually as the wavelength increased. The ∆pksP strain showed no characteristic peak depicting the absence of melanin.

**Fig. 2:**
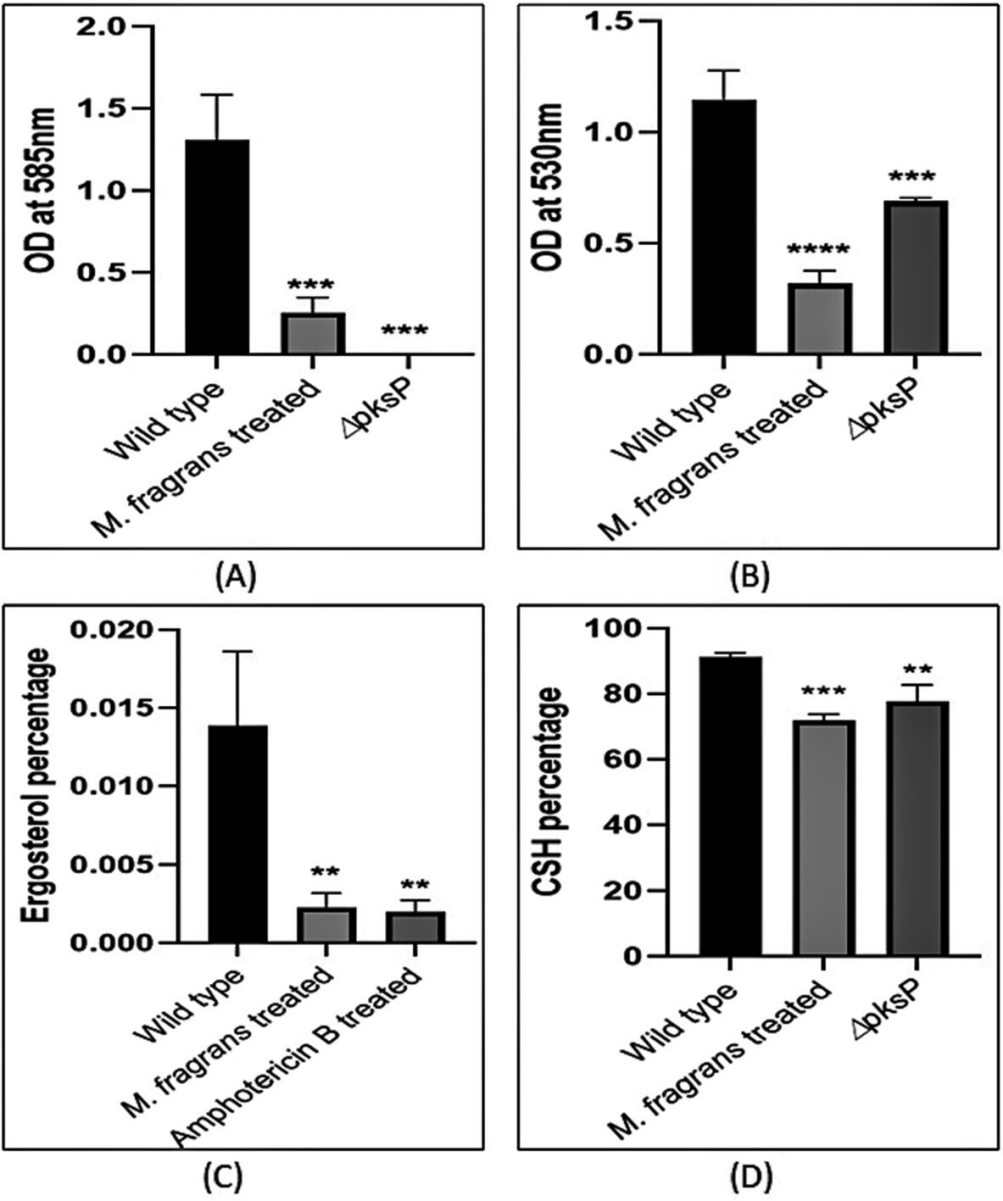
Evaluation of the physical properties of the conidial surface. The *∆pksP* strain (negative control) and wild type *A. fumigatus* with JaH extract 0.078 mg/mL, Amp B 0.012 mg/mL (drug control) and without any treatment (positive control) were grown on Czapek medium for 5 days at 28 °C. *A. fumigatus* extract treated culture showed reduced melanin content (**a**), decreased conidia formation (**b**), altered membrane surface due to reduction in ergosterol content (**c**) and cell surface hydrophobicity (**d**) analogous to the *∆pksP* strain and Amp B treated culture. *p* < 0.05 was considered statistically significant

The extracellular melanin content was estimated by spectrophotometric method at 585 nm. The results indicated reduction in melanin content after treatment as compared to the solvent control plates. Melanin content was reduced by 76.09% after treatment with the extract as compared to control whereas 99.8% reduction in the melanin content was estimated in ∆pksP culture (Fig. 2a; *p* < 0.05).

### Physical and chemical properties of melanin

**Fig. 3:**
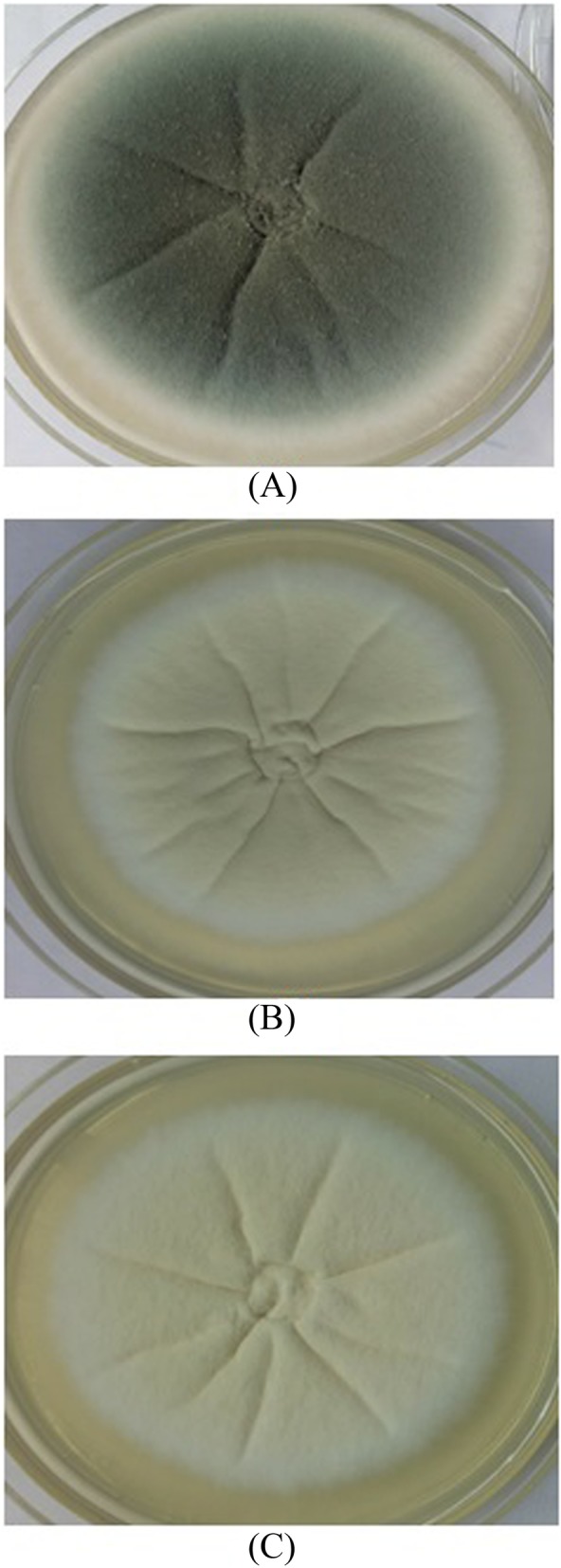
Effect of extract on *A. fumigatus* grown on Czapek medium. The wild type strain of *A. fumigatus* was grown on Czapek agar, supplemented (**b**) or not (**a**) with 0.078 mg/mL of JaH extract for 5 days at 28 °C. The colour of the colonies obtained in the presence of JaH is similar to that of colonies of *∆pksP* strain grown on Czapek medium (**c**)

The culture plates were phenotypically visualized and white colonies were observed after the extract treatment. Figure 3b shows the effect of *M. fragrans* extract on melanin inhibition in *A. fumigatus* at MEC as compared to wild type colonies (Fig. 3a) and ∆pksP (Fig. 3c). The extracted pigment was characterized on the basis of physico-chemical tests. The extracted pigment was soluble in NaOH and KOH whereas, insoluble in water or organic solvents like chloroform, ethyl acetate, alcohol and acetone. The pigment was decolorized by the oxidizing agents H2O2 and KMnO4 and precipitated by 1% FeCl3.

### Effect of JaH extract on conidia production in *A. fumigatus*

Conidia formation was spectrophotometrically measured at 530 nm. It was observed that Conidia formation reduced by 72.1% after treatment with the extract of *M. fragrans* whereas ∆pksP culture showed 39.78% conidial reduction (Fig. 2b; *p* < 0.05).

### Ergosterol content estimation

*A. fumigatus* hyphae were analyzed for the ergosterol content with and without extract. Amphotericin B (Amp B), a known inhibitor of ergosterol was used as positive control. A reduction of 83.63 and 85.02% was found in the ergosterol content after treatment with extract and Amp B, respectively in comparison to control group (Fig. 2c; *p* < 0.05).

### Cell surface hydrophobicity

CSH was assessed by two-phase partitioning with hexadecane as the hydrocarbon phase. A decrease in the CSH for both treated and ∆pksP culture was observed as compared to the control wild type greenish grey conidia. The treated conidia showed 72.20% decrease in CSH and ∆pksP culture exhibited 77.79% (Fig. 2d; *p* < 0.05) CSH reduction. This decrease in hydrophobicity was also observed during the preparation of conidial suspensions.

### Scanning electron microscopy

**Fig. 4:**
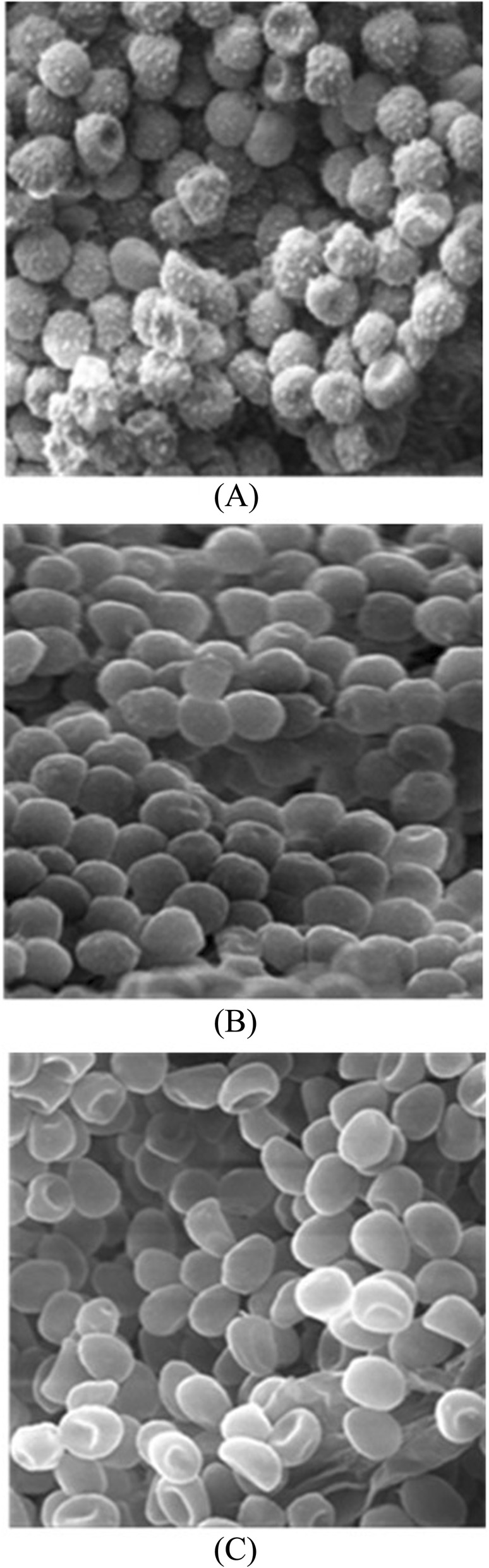
Visualisation of the conidial surface by scanning electron microscopy. Micrograph of conidia from 5- days- old cultures of wild type *A. fumigatus* (**a**), cultivated in the presence of JaH extract 0.078 mg/mL and ∆pksP strain (**c**). Magnification of (**a**) and (**b**) is 9.1 K X, of (**c**) is 5.6 K X and scale corresponds to 2 μm

The cell surface morphology of wild type conidia, ∆pksP and the extract treated *A. fumigatus* conidia were analyzed and compared by SEM. The wild type conidia showed echinulate surface. The ∆pksP revealed smooth conidial surface with complete absence of any protrusions. The extract treated conidia also revealed smooth conidial surface devoid of any ornamentation (Fig. 4a-c).

### Transmission electron microscopy

**Fig. 5:**
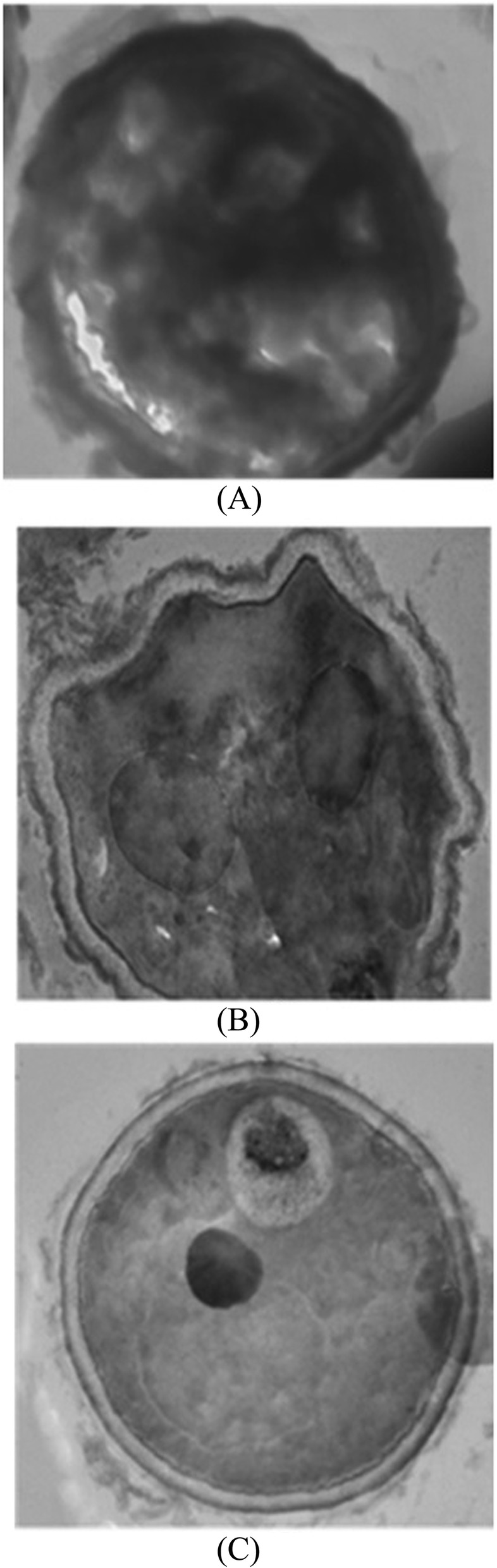
Ultrastructure of the conidial wall as visualised by transmission electron microscopy. Conidia from 5- days- old cultures of wild type *A. fumigatus* (**a**), cultivated in the presence of JaH extract 0.078 mg/mL and ∆pksP strain (**c**) were processed for ultra-structural examination of their cell wall. Conidia from wild type *A. fumigatus* shows dense layer around cell wall (**a**), whereas *A. fumigatus* cultivated in the presence of JaH extract (**b**) are relatively smooth surfaced similar to that of the ∆pksP strain (**c**)

Lateral section of the wild type, ∆pksP and treated conidia was visualized by TEM. The lateral section of the wild type conidia showed thick electron dense inner layer indicating the presence of melanin in between the membrane (Fig. 5a). In comparison to the wild type conidia, both ∆pksP and extract treated conidial section showed visible clear inner surface. There was an absence of electron dense layer. The two clear thin layers were also devoid of cell surface ornamentations (Fig. 5b-c).

### Chromatographic analysis

Forty-two compounds were characterized and identified according to their mass spectra and their relative retention indices determined on a non-polar stationary phase capillary column, comprising 97.1% of the total extract. The identified compounds are listed in Table 2 in elution order from the ZB-5 column, along with the percentage composition of each component and its refractive index. The main constituents were identified as sabinene (12.2%), linoleic acid (11.7%), hexadecanoic acid (10.5%), safrole (8.1%), elemicin (7.8%), myristicin (6.7%) and β-pinene (6.5%). The extract was found to be rich in monoterpene hydrocarbons (36.4%), followed by long chain oxygenated hydrocarbons (29.5%), phenylpropanoids (17.5%), oxygenated monoterpenes (10.9%), sesquiterpene hydrocarbons (2.4%), oxygenated diterpene (0.3%) and diterpene hydrocarbon (0.1%).

**Table 2:** Chemical constituents of JaH extract

Compound	RI	Area %	Mode of Identification
*α*-Thujene	897	1.4	RI, MS
*α*-Pinene	903	3.8	RI,MS, CI
α-Fenchene	913	0.3	RI, MS
Camphene	914	t	RI, MS
Sabinene	934	12.2	RI, MS
*β*-Pinene	937	6.5	RI, MS
Myrcene	947	1.7	RI, MS, CI
*α*-Phellandrene	960	1.8	RI, MS, CI
*δ*-3-Carene	966	0.5	RI, MS
*α*-Terpinene	972	0.8	RI, MS, CI
*o*-Cymene	979	0.3	RI, MS
Limonene	983	4.5	RI, MS, CI
1,8-Cineole	986	0.2	RI, MS. CI
*γ*-Terpinene	1013	1.1	RI, MS
*cis*-Sabinene hydrate	1023	0.7	RI, MS
Terpinolene	1045	1.5	RI, MS, CI
*trans*-Sabinene hydrate	1057	0.8	RI, MS
1,3,8-p-Menthatriene	1071	t	RI, MS
Terpin-4-ol	1148	0.8	RI, MS, CI
*α*-Terpineol	1163	0.3	RI, MS, CI
Sabinene hydrate acetate	1241	t	RI, MS
Safrole	1288	8.1	RI, MS
*δ*-Elemene	1354	0.3	RI, MS
Eugenol	1364	t	RI, MS, CI
*α*-Copaene	1386	0.3	RI, MS
*β*-Cubebene	1403	t	RI, MS
Methyl eugenol	1420	1.3	RI, MS, CI
*β*-Caryophyllene	1437	0.7	RI, MS, CI
(*E*)-Isoeugenol	1471	0.5	RI, MS, CI
*α*-Humulene	1477	0.7	RI, MS
*γ*-Muurolene	1503	t	RI, MS
Germacrene D	1508	0.4	RI, MS
(*E*)-Methyl isoeugenol	1527	0.8	RI, MS
Myristicin	1556	6.7	RI, MS, CI
Elemicin	1595	7.8	RI, MS
(*E*)-Isoelemicin	1695	0.4	RI, MS
Hexadecanoic acid	2003	10.5	RI, MS, CI
Ethyl hexadecanoate	2028	0.4	RI, MS
13-*epi*-manool oxide	2044	0.3	RI, MS
Kaurene	2065	0.1	RI, MS
Linoleic acid	2150	6.9	RI, MS
Oleic acid	2156	11.7	RI, MS
Monoterpene hydrocarbons		36.4	
Oxygenated monoterpenes		10.9	
Sesquiterpene hydrocarbons		2.4	
Diterpene hydrocarbon		0.1	
Oxygenated diterpene		0.3	
Phenylpropanoids		17.5	
Long chain oxygenated hydrocarbons		29.5	
Total identified		97.1	

*RI* retention index relative to C_8_-C_25_ *n*-alkanes on ZB-5 column, *MS* NIST and Wiley library and the literature, *CI* Co-injection of commercial samples, *t* trace (< 0.1%)

### Protein ligand interaction

**Fig. 6:**
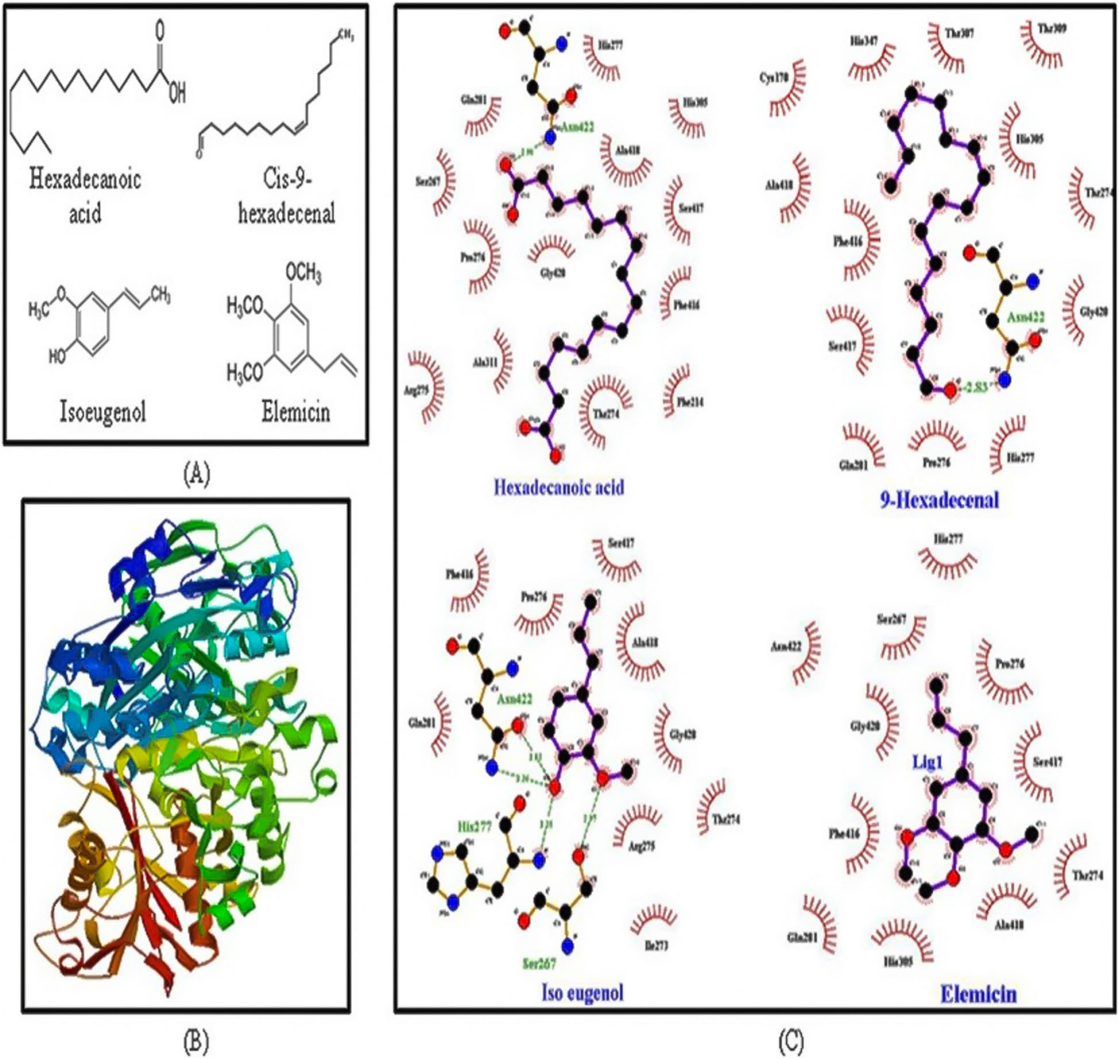
Docking study of the compounds with ketosynthase domain of polyketide synthase protein in *A. fumigatus*. **a** Structure of hexadecanoic acid, cis-9-hexadecenal, isoeugenol and elemicin, (**b**) structure of ketosynthase domain of polyketide synthase protein was retrived from Swiss-model, (**c**) protein- ligand interactions analysed by LigPlot. Dashed lines indicate hydrogen bonds between the atoms involved. The arc with spokes towards the ligand atoms shows the hydrophobic interaction. Hexadecanoic acid, cis-9-hexadecenal and isoeugenol form proper hydrogen bonding whereas elemicin shows hydrophobic interaction only

The LigPlot analysis depicted that despite of having low binding energy, elemicin (− 5.35 Kcal/mol) was stabilized solely by hydrophobic interaction and formed no hydrogen bond with the amino acid residues of KS domain of PKS protein. The lower binding energy was observed in hexadecanoic acid (− 4.19 Kcal/mol), its derivative compound cis-9-hexadecenal (− 5.71 Kcal/mol) and isoeugenol (− 6.14 Kcal/mol). They formed both hydrogen bonds and hydrophobic interactions with PKS. Hexadecanoic acid and cis-9-hexadecenal formed proper hydrogen bonding at Asn422 whereas in isoeugenol Ser267, His277 and Asn422 was contact residue within the docking site (Fig. 6c). Cross comparing the interaction revealed that all the test ligands interacted at Asn422 residue of KS domain of PKS.

## Discussion

Herbs and spices have been used as dietary supplements and traditional medicines since ages. Previous study by Mc Fadden et al. reported that crude spice extracts exhibit good antifungal activity and the probable mechanism involves the inhibition of various cellular processes, augmenting the membrane permeability and finally, leading to leakage of ions from the cells [37]. *M. fragrans* is a spice widely used as flavoring agent in food industries. In the present study, polarity guided extraction of *M. fragrans* was done using hexane, chloroform, methanol and ethanol based on their increasing polarity. It has been reported that the type of solvent used for extract preparation impacts the antimicrobial activity because even after the solvents were removed from extracts by evaporation; chemical compounds extracted using various solvents were different. In the present study JaH extract exhibited best result which coincides with the study reported by Witkowska et al. [12]. Hexane being non polar solvent breaks the hydrophobic barriers and hexane extract contains maximum lipophilic metabolites lignin, wax, lipids, sterols and terpenoids [38–40]. Thus, hexane extract was used for further studies.

The commonly reported phytochemical constituents of *M. fragrans* are volatile substances, terpenoids, phenolics, lignin compounds, protein, mucilage and starch [41]. The present study confirmed the presence of alkaloids, steroids, tannins and terpenoids in *M. fragrans* extract. However, Iyer et al. reported the absence of terpenoids in their extracts [42]. This variation may be attributed to the plant sources, climatic conditions and the extraction methodology [43].

While the phytochemical constituents of *M. fragrans* have been extensively studied for its antifungal effect [42, 44–47], the melanin inhibiting potential has not been studied till date. The present work aimed at evaluating the antifungal effect of *M. fragrans* extract as a melanin inhibitor against *A. fumigatus*. The role of DHN-melanin as an important virulent factor has been broadly described [1, 3, 20, 28]. It has also been reported that mutation in each gene of the DHN-melanin biosynthesis cluster produced different colored colony. *∆pksP A. fumigatus* produced white avirulent colonies, whereas deletion of other genes of DHN-melanin cluster produced grey, reddish pink and brown conidia [1]. Among all extracts used, JaH extract showed melanin inhibition at minimum concentration against *A. fumigatus*. The culture plates with JaH extract at MEC were seen to be white in color as compared to the control wild type culture which was greenish grey (Fig. 3). Hence, it was hypothesized that the target of the extract is either *pksP* gene or its translational product which remains to be clarified.

Melanin was extracted from *A. fumigatus* after JaH extract treatment as well as wild type culture. The extracted pigment showed positive result for the chemical tests used for fungal melanin diagnosis [28]. The result depicted that the absorbance declined as the wavelength increased to the visible region which is the property of aromatic organic compound. The result coincides with previous report by Raman and Ramasamy [48].

Melanin pigment plays an important role in cell surface morphology. The loss of pigment biosynthesis leads to extreme changes on the conidial wall orientation. Hence, to further study the effect of JaH extract on cell wall, ergosterol estimation was conducted. Sterols are neutral lipids of eukaryotic cells and play an essential role in the maintenance of cell membrane. Among sterols, ergosterol is the main component of fungal membrane which makes its biosynthetic pathway essential for fungal growth [49]. It is also the primary target for antifungal drug such as Amp B. A marked decrease in the ergosterol percentage was observed in treated conidia in comparison to the wild type conidia (Fig. 2c). The result indicated that JaH extract reduces the ergosterol production which may lead to changes in membrane fluidity, regulation and distribution of integral membrane proteins.

Cell surface hydrophobicity (CSH) contributes to the interaction between *A. fumigatus* and the host epithelial cell surface, which is an important factor for spreading infection. It has been reported that there is a direct relation between cell surface hydrophobicity and adherence to the host surface [50–52]. Hence, it can be concluded that decrease in cell surface hydrophobicity would result in decrease in adherence to the host cell surface. Melanin increases both negative charge and hydrophobicity of the conidia. The pigment-less conidia showed decrease in CSH as compared to the wild type conidia which suggests that blockage of the melanin biosynthetic pathway leads to the reduction of some hydrophobic components on the conidial surface contributing to the marked loss of adherence properties of the conidia. Similar results were reported on the *∆pksP* strain by Pihet et al. [20].

Melanized conidia have rough outer layer with protrusions on the surface. These conidial protrusions protect the organism from phagocytosis and increase its resistance to ROS produced by phagocytic cells. In the present study, the cell surface morphology of conidia was analyzed using SEM, which clearly indicated loss in cell surface protrusions and showed smooth cell wall conidia in the treated fungus. TEM examination also confirmed protrusion less surface, thinner cell wall and reduced melanin in treated conidia in comparison to control probably due to the progressive detachment of the outermost cell wall layer. Similar results were reported by Jahn et al. and Youngchim et al. [1, 53].

The GC-MS analysis of the JaH extract revealed the presence of sabinene, linoleic acid, hexadecanoic acid, safrole, elemicin, myristicin and *β*-pinene in varying quantities. Major constituent in essential oil of *M. fragrans* are myristicin, safrole, pinene, isoeugenol, 4-terpenol as reported by Alcazar-Fuoli and Mellado [54]. Various studies have also reported the presence of neolignan and macelignan in *M. fragrans* extract [14, 42]. The quantitative and qualitative divergence of plant constituents may be due to the geographical, climatic, and soil conditions, which in turn may affect the composition and/or synthesize new secondary metabolites from the same plant species [55]. The potential role of these components needs to be further analyzed.

Ligand-protein interactions can be extrapolated through docking studies. Polyketide synthase is an essential protein for synthesis of conidial pigment melanin. *A. fumigatus* PKS protein contains three important domains: ketosynthase (KS), acyltransferase (AT) and acyl carrier protein (ACP). KS domain is the functionally active site of the enzyme [56]. The KS accepts the acyl unit from the acyl-ACP and condenses malonyl-CoA units shuttled by the ACP onto the starter unit to add a ketide unit [57]. In the present study, structure of KS domain of PKSs protein of *A. fumigatus* was modelled for the docking studies with the compounds. The structural differences among the ligands may contribute to differences in the interaction [58]. Based on LigPlot analysis, the O atom located on the hydrocarbon chain was situated to form H-bonds with Asn422 in the ligands. By contrast, the structural difference in elemicin pushes the same O atom away from the H-bond forming amino acids, resulting hydrophobic interactions only (Fig. 6c).

## Conclusion

The study concludes that the JaH extract of *M. fragrans* has potential antimelanogenic properties as depicted by inhibition of melanin synthesis, loss in cell surface protrusions, formation of smooth cell wall, reduction in ergosterol concentration and cell surface hydrophobicity. Thus, it can potentially decrease the pathogenicity of *A. fumigatus* and increase its susceptibility to available antifungal drugs. Hence, *M. fragrans* extract in combination with commonly used antifungals may increase the therapeutic efficacy. This combination approach may be helpful for large number of patients suffering with *A. fumigatus* infections.

## Data Availability

The datasets used and/or analyzed during the current study will be made available from the corresponding author on reasonable request.

## Abbreviations

*∆pksP*: Polyketide synthase gene mutant strain
ABPA: Allergic bronchopulmonary aspergillosis
ACP: Acyl carrier protein
Amp B: Amphotericin B
AT: Acyltransferase
CLSI: Clinical and Laboratory Standards Institute
CSH: Cell Surface Hydrophobicity
CzA: Czapek Dox Agar
CzB: Czapek Dox Broth
DHN: 1, 8-dihydroxy naphthalene
DMSO: Dimethyl sulfoxide:
FID: Flame ionization detector
GC–MS: Gas chromatography–mass spectrometer
JaC: Chloroform extract of *Myristica fragrans*
JaE: Ethanol extract of *Myristica fragrans*
JaH: Hexane extract of *Myristica fragrans*
JaM: Methanol extract of *Myristica fragrans*
KS: Ketosynthase
MEC: Minimum Effective Concentration
PBS: Phosphate buffer saline
PKS: Polyketide synthase
RI: Retention index
ROS: Reactive oxygen species
SEM: Scanning Electron Microscopy
TEM: Transmission Electron Microscopy
